# A Systematic Review Protocol to Determine the Most Effective Strategies to Reduce Computed Tomography Usage in the Emergency Department

**DOI:** 10.7759/cureus.9509

**Published:** 2020-08-01

**Authors:** Jason L Elzinga, Cody L Dunne, Allen Vorobeichik, Diana Keto-Lambert, Daniel Grigat, Eddy Lang, Shawn Dowling

**Affiliations:** 1 Emergency Medicine, University of Calgary, Calgary, CAN; 2 Pediatrics, University of Alberta Evidence-Based Practice Center, Edmonton, CAN; 3 Emergency Medicine, Strategic Clinical Network, Alberta Health Services, Calgary, CAN; 4 Emergency Medicine, Alberta Health Services, Calgary, CAN

**Keywords:** emergency department, ct use, computed tomography, systematic review, meta-analysis, reduction intervention, radiography

## Abstract

This study describes the protocol for a systematic review and meta-analysis. The primary objective of the review is to identify experimental studies assessing the effectiveness of interventions that aim to reduce the proportion of computed tomography (CT) in emergency departments (EDs). Data permitting, our secondary objectives will be to assess the impact of reduction in CT utilization on the length of stay, admission to hospital, and uptake/satisfaction with the intervention. When available, balancing measures such as readmission to hospital or ED revisit rates will be included. Pre-defined subgroup analyses include patient populations (adult or pediatric), type of ED, and the nature of the intervention.

Through this review, the research team aims to inform knowledge translation initiatives aimed at lowering CT usage in the ED by identifying the most effective interventions to safely improve CT resource stewardship.

## Introduction

Description of the issue

Computed tomography (CT) is an important diagnostic tool used in the assessment of patients in the Emergency Department (ED). However, emerging evidence has shown dramatic escalations in CT utilization for adult ED patients, with reported annual increases of 6.0%-13.8% [1,2]. While CT is an important diagnostic imaging modality, it is also associated with radiation exposure, increased health system costs, longer ED lengths of stay, and impaired flow of patients through the department [3]. Data suggests that national CT utilization for ED patients is increasing at rates exceeding the increase in patient volumes. Some of these CTs are likely avoidable. As an example, one study looking at low-risk headaches suggested that more than 38% of patients may be imaged unnecessarily [4]. If the increasing use of CT was associated with improved patient-oriented outcomes, the risks may be worth the benefits but that is not always the case. As an example, the use of CT pulmonary angiograms to diagnose pulmonary emboli (PE) has been steadily rising but the rates of massive PEs and the mortality rate due to them have been on the decline [5-7].

Considering the risks of CT imaging, reducing the rate of non-urgent CTs is an important target for improving care and, as a result, has been identified as a priority in many health systems. A number of studies have attempted to develop clinical decision rules (CDRs) to safely forego advanced imaging for patients with certain clinical conditions. However, there is a fundamental difference between developing a sensitive and well-validated CDR and successfully implementing one to change clinical practice. Despite having validated CDRs for head and neck trauma, acute headache, and pulmonary embolism, as well as potential alternatives to CT imaging for many presentations, these studies have clearly not translated clinically to a reduction in the overall CT usage. Therefore, this review will focus on the knowledge translation aspect of practically implementing interventions that will lead to a reduction in ED CT utilization.

Description of the existing intervention data

Interventions aimed at decreasing unnecessary CT utilization can be grouped into three broad categories: pre-ordering, at the time of ordering, and post-imaging feedback [8-10]. Assessing these strategies and their effectiveness at reducing imaging is a popular topic in the literature with several systematic reviews having been published. However, this literature frequently focuses on specific pathologies (e.g., CT use in acute low-back pain patients), includes multiple imaging modalities, or reviews the incidence of low-value testing in general without addressing potential solutions [7,10-15].

Furthermore, the data that can be extrapolated to the ED setting from these prior reviews is limited. Of the current knowledge translation literature, one review aimed at reducing CT use in low-back pain found targeted reminders of indications and a modified referral form with decision support reduced CT usage by 36.8% and 22.5%, respectively. The same study found no benefit from physician audits or feedback tools, education sessions, or guidelines dissemination [14]. However, this study included both outpatients and all hospitalized patients with acute and chronic pain, so its generalizability to the ED setting is limited. In contrast, a review in the pediatric population found that patient/family education was the single most effective intervention with a relative reduction of 57.9%, and that multi-faceted interventions were more effective than single focused ones [15].

Rationale

A review of the literature demonstrates that while there have been several individual and heterogeneous studies attempting to reduce CT utilization, there has not been an effective review to aggregate this data and make generalizable conclusions to guide quality improvement efforts. The limited data available is not specific to the ED population and presents contradictory evidence. This suggests that both the patient population and the clinical setting likely impact the effectiveness of interventions.

Through this systematic review, the authors aim to identify and summarize the literature available regarding the effectiveness of various interventions to reduce CT usage in the ED. We will also explore factors that predict the effectiveness of interventions based on *a priori* defined subgroups (e.g., the nature of the intervention, whether the intervention was co-designed by providers, whether it was multi-faceted, etc.). By identifying those interventions that have had the most significant impacts, targeted strategies can be designed to ensure physicians are ordering CTs in such a way that maintains timely access to CT while ensuring patient safety and resource stewardship.

## Materials and methods

Objectives

Through this systematic review, the authors’ primary objective is to identify and summarize the literature available on the effectiveness of various interventions to reduce CT usage in the ED.

When data is available, we will assess a variety of secondary outcomes that include the association between reduction in CT usage and ED length of stay, admission to hospital, and satisfaction/uptake of the intervention. Additionally, balancing measures such as readmission to hospital or ED revisit rates will be captured when the data is presented in individual studies.

Secondary objectives will include the exploration of predictors of effective interventions based on *a priori* defined subgroups: target population (adult versus paediatric, or both), study context (academic versus community centre, country of the study), nature of the intervention (implementation of clinical decision rule, education-only focused, policy, or workflow changes), and single versus multi-faceted intervention.

Study inclusion factors

The various factors taken into consideration for the inclusion of studies are described in Table 1.

**Table 1:** PICOST criteria for the inclusion of studies into the systematic review and meta-analysis CT, computed tomography; ED, emergency department; PICOST, Population, Intervention, Control, Outcome(s), Study Design(s), Timing

Criteria	Description
Participants	All ED patients undergoing CT (all modalities). Adult and pediatric patients will be included
Intervention	ED-based intervention to reduce low-value CT utilization
Control	Depending on the experimental design, either concurrent or historic groups not receiving the intervention
Primary outcome measures	Reduction in the proportion of CTs ordering (normalized to ED census where possible)
Secondary outcome measures	Physician utilization of implementation strategy, ED length of stay, diagnostic yield of CTs, economic impact of reduction in imaging, stratification by CT modality/acuity/indication, ED revisit/readmission rates, admission rates, physician and patient satisfaction scores
Study designs	Randomized and non-randomized clinical trials including clinical controlled trials, interrupted time series and before-after trials
Timing	From database inception to April 27, 2020

Search methods

Our search methodology was developed and piloted by a librarian/information specialist (DKL) in partnership with the broader review team and peer-reviewed by another librarian.

Electronic Search 

An initial search strategy was developed for Ovid MEDLINE® using a combination of key term words and Medical Subject Headings (MeSH) terms. Key terms in three concept areas were utilized: emergency medicine (e.g., "Emergency Medicine", "Trauma Centers", "Critical Care", etc.), diagnostic imaging (e.g., "CT Scan", "Radiography", "Neuroimaging", etc.), and reduction practices (e.g., "Overuse", "Health Care Costs", "Unnecessary Procedures", etc.).

To ensure the strategy identified relevant articles, a short literature review was conducted *a priori* (EL) that included several notable studies in this area. The results of the strategy were checked to ensure all studies were captured in our search.

The search strategy was then adapted for several other databases, including Embase (Ovid), CINAHL (EBSCO), and Cochrane Central Register of Controlled Trials (CENTRAL). Appendix 1 outlines the search strategy used for MEDLINE (Ovid).

Grey Literature

Grey literature search strategies included ProQuest Dissertation & Theses Global™, conference proceedings, Google Scholar web search (including the first 10 pages of results), contacting experts in the field for unpublished and ongoing trials, and contacting study authors for additional information and unpublished results, if necessary. Additionally, the bibliography of the searches retrieved from the electronic search were reviewed and forward reference searching of cited articles was also conducted.

## Results

Study selection and quality assessment

**Figure 1:**
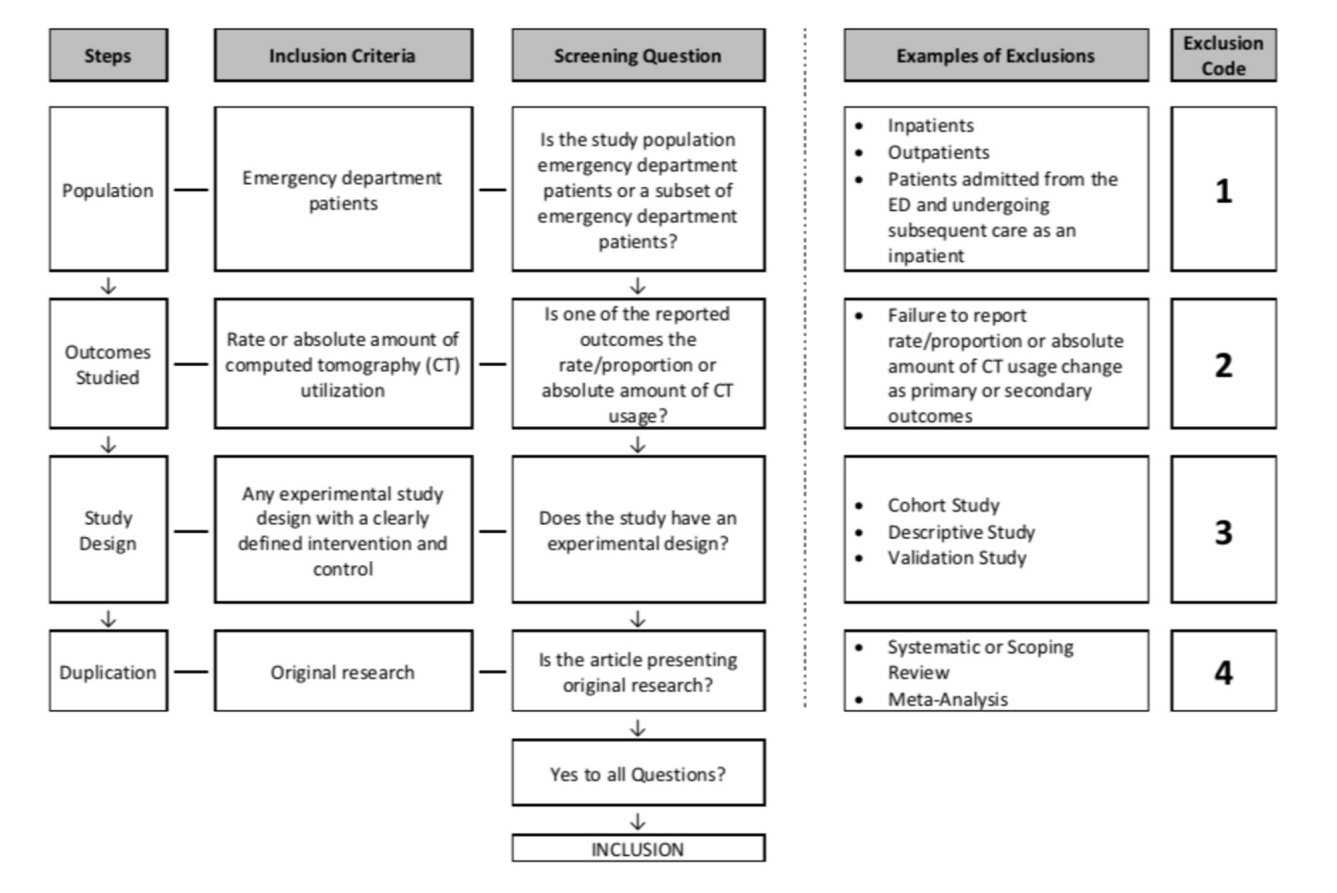
Standardized tool utilized by reviewers for article selection CT, computed tomography; ED, emergency department

All identified studies will be imported into Covidence systematic review software (Veritas Health Innovation Ltd, Melbourne). A standardized tool (Figure 1) has been developed and piloted by the authors and will be used to aid in the selection of studies.

Each title and abstract will be screened by two independent reviewers to identify potential articles and remove any duplicates. Following this, a full text review of applicable studies will be conducted by two independent reviewers who will apply the eligibility criteria to each study using a standardized form. In cases of disagreement, a third reviewer will adjudicate and reach a final decision on inclusion. This strategy will be used to limit the risk of selection bias.

Any excluded articles will have a reason captured and reported. This information will be presented in the final report in the form of a Preferred Reporting Items for Systematic Reviews and Meta-Analyses (PRISMA) diagram [16].

Two independent reviewers will also conduct a formal risk of bias assessment. The tool used will be based on the nature of the study (i.e., randomized or non-randomized/observational); however, either will be validated and supported by the Cochrane Collaboration [17-18]. Any discrepancies in risk of bias assessment will be decided upon by a third reviewer. We will be employing the Grading of Recommendations Assessment, Development and Evaluation (GRADE) methodology for certainty of evidence relating to the primary outcome where applicable.

Data extraction and analysis

Data extraction will occur by two separate investigators in duplicate using a standardized data extraction form. A third member of the team will adjudicate any discrepancies.

Descriptive statistics and narrative analysis will be used to summarize the data. Heterogeneity of the data (for the primary and secondary outcomes) will be measured by I^2^ while publication bias will be assessed using a funnel plot if more than 10 studies are included in the analysis. When possible, we will pool the results and undertake a meta-analysis utilizing a random effects model using the Review Manager Version 5.3 (Nordic Cochrane Centre, Cochrane Collaboration, Copenhagen). Data will be separated by study design with dichotomous data reported using risk ratios and continuous data reported as mean difference, both with 95% confidence intervals.

Subgroup and Sensitivity Analysis

*A priori* subgroups have been defined. These include target population (adult versus paediatric, or both), study context (academic versus community centre, country of the study), nature of the intervention (Implementation of clinical decision rule, education-only focused, policy, or workflow changes), and single versus multi-faceted intervention. The statistical approach for subgroups will be the same as for the primary outcome.

The effect of methodological design on summary estimates of sensitivity will be assessed. If enough studies are included, sensitivity analysis will consider whether studies were design for research or quality improvement.

## Discussion

Systematic versus scoping review

Scoping reviews can be useful for identifying current and emerging evidence in a topic area, while systematic reviews seek to rigorously appraise and synthesize the evidence base on a highly specific question to inform policy, practice, and further research [19]. While this is a broad topic in its nature, and simply identifying what strategies exist in the current literature may lend itself to a scoping review design, emergency medicine practitioners are already aware of the multitude of strategies employed by ED leadership, hospital administration, and government to limit the overuse of imaging. The implementation of these strategies, however, has resulted in mixed results on the their effectiveness. For example, efforts exist to reduce low-value CT imaging for specific conditions such as low back pain and cervical spine injuries, and there are a number of clinical decision rules for CT use in the ED [20-22]. However, these studies have reported mixed results in terms of impact, and there is a lack of clarity about which specific implementation strategies are most effective for achieving reductions in low-value CT imaging.

As researchers specifically interested in knowledge translation, our objective is to identify the strategies that are most effective for achieving clinical practice change in the ED. A systematic review will allow us to not only identify which strategies have been studied, but may also allow us to determine, with our sensitivity analysis, what setting and what conditions may contribute to the effectiveness of interventions.

Potential impact for emergency medicine

Resource stewardship and improving resource usage has been a focus of many campaigns over the past decade. Choosing Wisely Canada (CWC) and the Canadian Association of Emergency Physicians (CAEP) have partnered to create 10 recommendations where this may be possible in the ED. Six of these directly focus on reducing unnecessary image ordering [23]. Similarly, the American College of Emergency Physicians (ACEP) has released a position statement on the topic [24]. This highlights the need for improvement in how we decide to use decision supports and other tools that have already been validated and readily disseminated.

Safely decreasing CT usage in the ED will have an important impact on our patients, our department, and our health care system. From a system accessibility perspective, reducing low-value CTs will increase ED capacity as patients undergoing CT experience increased ED length-of-stays and inhibit access to the CT for other ED patients who have an urgent/emergent indication. CWC states that as many as 30% of tests, treatments, and procedures in Canada are potentially unnecessary, and that reducing inappropriate tests can save money, reduce wait times, and improve patient experience [25]. From a patient perspective, reducing CT utilization will decrease their length of stay in the ED, decrease their exposure to ionizing radiation, and reduce the risk of harm from intervention/investigation of incidental findings (potentially leading to over-diagnosis and over-treatment). Finally, given the current constraints of the health care system, reducing health care costs is an important consideration, especially in light of the aforementioned benefits.

However, more data is needed to help translate successes from narrowly targeted studies to interventions that are globally effective. Table 2 identifies several of the strategies that have previously been suggested as interventions to achieve the goal of CT utilization reduction. This review aims to further inform these strategies as the identification and assessment of the available literature is completed.

**Table 2:** Categorized examples of interventions Interventions that have been implemented in an effort to reduce unnecessary CT usage [5-7]. CT, computed tomography

Time	Examples of intervention
Pre-ordering	Access to patient’s prior imaging records, education regarding practice guidelines and equivalent imaging, patient education regarding benefits versus risk, imaging facility accreditation
At the time of ordering	Requiring pre-authorization for imaging (e.g., by radiologist or insurer), displaying cost of imaging, clinical decision support built into requisition, duplicated study notification
Post-imaging	Audit and feedback reports to ordering physicians on their CT usage rates, image reports inclusion of appropriate clinical practice guidelines

## Conclusions

This report describes the systematic review and potential meta-analysis protocol that will be utilized to determine and assess the evidence available regarding the effectiveness of various interventions at reducing CT usage in emergency departments. The results of this review will help inform ED leadership, hospital administration, and governments looking to balance patient safety with resource stewardship as they implement strategies to reduce the proportion of low-value CTs.

## Appendices

Appendix 1

**Table 3:** Search strategy design based on the MEDLINE (Ovid) format

#	Searches
1	Emergency Treatment/
2	Emergency Medicine/
3	Emergency Medical Services/
4	Emergency Service, Hospital/
5	Trauma Centers/
6	Triage/
7	Evidence-Based Emergency Medicine/
8	Emergency Nursing/
9	Emergencies/
10	(emergicent* or emerge-cent*).tw,kf.
11	casualty department*.tw,kf.
12	((emergenc* or ED or ER) adj3 (room* or accident$1 or ward or wards or unit or units or department* or physician* or doctor* or nurs* or treatment* or visit* or personnel or staff or service*)).tw,kf.
13	(triage or critical care or acute-care or (trauma adj1 (cent* or care))).tw,kf.
14	or/1-13 [POPULATION - EMERGENCY DEPARTMENT PATIENTS]
15	Diagnostic Imaging/
16	exp Tomography, X-Ray Computed/
17	Neuroimaging/
18	(radiograph* or radio-graph* or radiation).tw,kf.
19	(Xray* or X-ray*).tw,kf.
20	(CT scan* or CT-exam* or CAT scan* or (comput* adj2 tomograph*)).tw,kf.
21	(image* or imaging*).tw,kf.
22	(neuroimag* or neuro-imag*).tw,kf.
23	or/15-22 [MeSH & KEYWORDS FOR CT SCANS]
24	exp Health Services Misuse/
25	Unnecessary Procedures/
26	Guideline Adherence/
27	exp Decision Support Techniques/
28	Health Care Costs/
29	Cost Control/
30	(CT head rule* or CCHR).tw,kf.
31	(overdiagnos* or over-diagnos*).tw,kf.
32	(overtreat* or over-treat*).tw,kf.
33	(overuse* or over-use*).tw,kf.
34	(overutiliz* or over-utiliz*).tw,kf.
35	(overscan* or over-scan*).tw,kf.
36	((unnecessar* or ineffectiv* or decreas* or reduc*) adj3 (use* or usage* or utiliz* or test? or testing or scan* or imag* or screen* or radiation)).tw,kf.
37	or/24-36 [MeSH & KEYWORDS FOR REDUCTION PRACTICES]
38	14 and 23 and 37

## References

[1] Berdahl CT, Vermeulen MJ, Larson DB, Schull MJ (2013). Emergency department computed tomography utilization in the United States and Canada. Ann Emerg Med.

[2] Bellolio MF, Heien HC, Sangaralingham LR (2017). Increased computed tomography utilization in the emergency department and its association with hospital admission. West J Emerg Med.

[3] Perotte R, Lewin GO, Tambe U (2018). Improving emergency department flow: reducing turnaround time for emergent CT scans. AMIA Annu Symp Proc.

[4] Mackenzie MJ, Hiranandani R, Wang D, Fung T, Lang E (2017). Determinants of computed tomography head scan ordering for patients with low-risk headache in the emergency department. Cureus.

[5] Smith SB, Geske JB, Kathuria P (2016). Analysis of national trends in admissions for pulmonary embolism. Chest.

[6] Soylemez Wiener R, Schwartz LM, Woloshin S (2012). Time trends in pulmonary embolism in the United States: evidence of overdiagnosis. Arch Intern Med.

[7] Deblois S, Chartrand-Lefebvre C, Toporowicz K, Chen Z, Lepanto L (2018). Interventions to reduce the overuse of imaging for pulmonary embolism: a systematic review. J Hosp Med.

[8] Bernardy M, Ullrich CG, Rawson JV (2009). Strategies for managing imaging utilization. J Am Coll Radiol.

[9] Litkowski PE, Smetana GW, Ziedel ML, Blanchard MS (2016). Curbing the urge to image. Am J Med.

[10] Colla CH, Mainor AJ, Hargreaves C, Sequist T, Morden N (2017). Interventions aimed at reducing use of low-value health services: a systematic review. Med Care Res Rev.

[11] Viau JA, Chaudry H, Hannigan A, Boutet M, Mukarram M, Thiruganasambandamoorthy V (2019). The yield of computed tomography of the head among patients presenting with syncope: a systematic review. Acad Emerg Med.

[12] Ohana O, Soffer S, Zimlichman E, Klang E (2018). Overuse of CT and MRI in paediatric emergency departments. Br J Radiol.

[13] Tung M, Sharma R, Hinson JS, Nothelle S, Pannikottu J, Segal JB (2018). Factors associated with imaging overuse in the emergency department: a systematic review. Am J Emerg Med.

[14] Jenkins HJ, Hancock MJ, French SD, Maher CG, Engel RM, Magnussen JS (2014). Effectiveness of interventions designed to reduce the use of imaging for low-back pain: a systematic review. Can Med Assoc.

[15] Hiscock H, Neely RJ, Warren H, Soon J, Georgiou A. (2018). Reducing unnecessary imaging and pathology tests: a systematic review. Pediatrics.

[16] Moher D, Liberati A, Tetzlaff J, Altman DG, PRISMA Group (2009). Preferred reporting items for systematic reviews and meta-analyses: the PRISMA statement. PLoS Med.

[17] Sterne JAC, Hernán MA, Reeves BC (2016). ROBINS-I: a tool for assessing risk of bias in non-randomized studies of interventions. BMJ.

[18] The Cochrane Collaboration (2019). Cochrane Handbook for Systematic Reviews of Interventions version 6.0 (updated July. Cochrane Handbook for Systematic Reviews of Interventions Version 6.0.

[19] Munn Z, Peters MDJ, Stern C, Tufanaru C, McArthur A, Aromataris E (2018). Systematic review or scoping review? Guidance for authors when choosing between a systematic or scoping review approach. BMC Med Res Methodol.

[20] Liu C, Desai S, Krebs LD, Kirkland SW, Keto‐Lambert D, Rowe BH, for the PRIHS‐2 Choosing Wisely Team (2018). Effectiveness of interventions to decrease image ordering for low back pain presentations in the emergency department: a systematic review. Acad Emerg Med.

[21] Stiell IG, Wells GA, Vandemheen K (2001). The Canadian CT head rule for patients with minor head injury. Lancet.

[22] Desai S, Liu C, Kirkland SW, Krebs LD, Keto-Lambert D, Rowe BH (2018). Effectiveness of implementing evidence-based interventions to reduce C-spine image ordering in the emergency department: a systematic review. Acad Emerg Med.

[23] (2020). Emergency medicine. Ten things physicians and patients should question, by
Canadian Association of Emergency Physicians. https://choosingwiselycanada.org/wp-content/uploads/2017/02/Emergency-Medicine.pdf.

[24] American College of Emergency Physicians (2020). American College of Emergency Physicians. Resource utilization in the emergency department: the duty of stewardship. Policy resource and education paper (PREP). https://www.acep.org/globalassets/new-pdfs/preps/resource-utilization-in-the-emergency-department---the-duty-of-stewardship---prep.pdf.

[25] Canadian Institute for Health Information (2020). Canadian Institute for Health Information. Unnecessary care in Canada. Unnecessary care in Canada.

